# Palmitic Acid, A Critical Metabolite, Aggravates Cellular Senescence Through Reactive Oxygen Species Generation in Kawasaki Disease

**DOI:** 10.3389/fphar.2022.809157

**Published:** 2022-03-23

**Authors:** Qiongjun Zhu, Qianqian Dong, Xuliang Wang, Tianhe Xia, Yu Fu, Qiaoyu Wang, Rongzhou Wu, Tingting Wu

**Affiliations:** Children’s Heart Center, The Second Affiliated Hospital and Yuying Children’s Hospital, Institute of Cardiovascular Development and Translational Medicine, Wenzhou Medical University, Zhejiang, China

**Keywords:** kawasaki disease, coronary disease, metabolomics, cellular senescence, oxidative stress

## Abstract

Coronary artery lesions (CALs) are severe complications of Kawasaki disease (KD), resulting in stenosis and thrombogenesis. Metabolomic profiling of patients’ plasma could assist in elucidating the pathogenesis of CALs and identifying diagnostic biomarkers, which are imperative for clinical treatment. The metabolic profiles between KD patients with CALs and without CALs (non-coronary artery lesion, or NCAL, group) indicated the most significantly differentially expressed metabolite, palmitic acid (PA), showed the most massive fold change at 9.879. Furthermore, PA was proven to aggravate endothelial cellular senescence by increasing the generation of reactive oxygen species (ROS) in KD, and those two phenotypes were confirmed to be enriched among the differentially expressed genes between KD and normal samples from GEO datasets. Collectively, our findings indicate that cellular senescence may be one of the mechanisms of vascular endothelial damage in KD. PA may be a biomarker and potential therapeutic target for predicting the occurrence of CALs in KD patients. All things considered, our findings confirm that plasma metabolomics was able to identify promising biomarkers and potential pathogenesis mechanisms in KD. To conclude, Palmitic acid could be a novel target in future studies of CALs in patients with KD.

## Introduction

Kawasaki disease (KD) is a febrile, self-limited illness that mainly affects children <5 years and has become the most common cause of acquired heart diseases in children from developed countries (McCrindle et al., 2017). KD induces acute vasculitis of medium-sized arteries, especially coronary arteries, in multiple organs and tissues. During the acute phase of KD, coronary artery lesions (CALs) can develop and induce arterial wall damage and hemodynamic disorders. Up to 25% of patients without treatment will develop coronary artery aneurysms, and children with KD and CALs have a high risk of developing ischemic cardiomyopathy as adults (Kato et al., 1996). Recently, a retrospective analysis by Verdoni et al. (2020) proved that the incidence of Kawasaki disease among children has surged in areas widely affected by the coronavirus SARS-CoV-2.

However, the pathogenesis and mechanism of CALs development in KD patients remain unknown. Due to the geographical differences indicating higher susceptibility in Asian children, numerous researches have shown the critical roles genetics played by genetics in disease susceptibility. Other than that, innate immune response, matrix metalloproteinases, microRNAs, and IL-1 signaling pathways are hypothesized to be the cellular and molecular circuitries associated with disease progression (Noval Rivas and Arditi, 2020). At present, several clinical prospective studies suggest that the lipid distribution of KD patients with CALs is atypical (Gopalan et al., 2018; Shao et al., 2021; Zhang et al., 2021), accompanied by endothelial dysfunction and low-grade inflammation similar to atherosclerosis, leading to the development of atherosclerosis in adulthood (Senzaki et al., 2005; Mitani et al., 2009). Therefore, we postulate that metabolic disorders should play an essential role in the pathogenesis of KD with CALs.

Metabolomic technologies such as ultra-high-performance liquid chromatography- mass spectrometry can detect and semi-quantitatively measure hundreds of distinct metabolites. Data regarding differentially expressed metabolites can be used to explore metabolic pathways. These approaches have been used to identify physiological and metabolic profiles of different disease states and meaningful diagnostic biomarkers and provide new insights into disease studies (Fan et al., 2016; Gao et al., 2017; Rhodes et al., 2017; Mayerle et al., 2018). So far, metabolic studies have not been undertaken in KD. Therefore, we hypothesized that specific metabolites might be differentially expressed in KD patients with CALs than those without CALs (non-coronary artery lesion, or NCAL, group). These metabolites could disrupt metabolic pathways, leading to enhanced coronary arterial damage. Herein, we analyzed the plasma metabolic profiles of 40 KD patients with CALs (the CAL group) and 39 KD patients without CALs (the NCAL group) using ultra-high-performance liquid tandem chromatography quadrupole time-of-flight mass spectrometry (UHPLC-QTOFMS). Specific metabolic pathways were found to be differentially regulated between the two groups. A panel of 4 metabolites possessed sufficient power to distinguish between the CAL and NCAL groups in the acute phase of KD. The significant differentially expressed metabolites, such as palmitic acid, could provide novel biomarkers of diagnosing CALs early in the acute phase to compensate for the uncertainty of echocardiography currently used (Newburger et al., 2004).

As one of the crucial components of triglycerides in adipose tissue, PA maintains excellent clinical diagnostic and prognostic values (Katsareli and Dedoussis, 2014; Hui et al., 2019). Actually, excessive intake of PA will increase the risk of cardiovascular diseases (Harvey et al., 2010; Shen et al., 2013). According to transcriptomics and lipidomics studies, the expression level of lipid-regulating genes and lipid levels are significantly upregulated in senescent cells. Moreover, lipid regulation has also been proven to be the core of the cellular senescence procession (Saitou et al., 2018). We estimate that palmitic acid may as well exacerbate the CALs in KD patients by triggering cellular senescence.

Overall, we further explored the probable mechanisms of the differentially expressed metabolite palmitic acid in children with KD complicated with CALs based on metabolomics to confirm the predictive value of PA as a molecular marker.

## Methods

### Patients and Study Design

All patients were enrolled at the Second Affiliated Hospital and Yuying Children’s Hospital of Wenzhou Medical University (Zhejiang, China) between November 2014 and April 2016. Every patient and their parents received detailed information about the research and signed the consent form. This study was approved by the Ethics Committee of the Second Affiliated Hospital and Yuying Children’s Hospital of Wenzhou Medical University. All patients were clinically diagnosed as KD based on the American Academy of Pediatrics and the 2004 American Heart Association criteria (Newburger et al., 2004). Cardiac ultrasonography for heart function and coronary artery diameter measurement, and hematological examinations by laboratory tests were performed prior to intravenous immunoglobulin (IVIG) treatment and 7 days after IVIG treatment. CALs were confirmed using the following criteria (Newburger et al., 2004; Ronai et al., 2016): Z score of left anterior descending or right coronary artery≥2.5 was diagnosed as CALs.

Blood samples were collected in EDTA vacutainer tubes on day 7 after IVIG treatment, immediately placed on ice, and centrifuged (1000 g, 15 min) within 30 min. The supernatant was extracted and stored in a sterile tube at −80°C until required.

### Metabolomics Study

LC-MS/MS analyses were performed using an UHPLC system (1290, Agilent Technologies) with an UPLC BEH Amide column (1.7 μm, 2.1*100 mm, Waters) coupled to a TripleTOF 5600 (Q-TOF, AB Sciex). The mobile phase consisted of 25 mM NH4OAc and 25 mM NH4OH in water (pH = 9.75) (A) and acetonitrile (B), and an elution gradient was applied as follows: 0 min, 95% B; 7 min, 65% B; 9 min, 40% B; 9.1 min, 95% B; and 12 min, 95% B The flow rate was 0.5 ml min^−1^. The injection volume was 3 µL in the positive mode and 4 µL in the negative mode. The Triple TOF mass spectrometer was used for its ability to acquire MS/MS spectra on an information-dependent basis (IDA) during the LC/MS experiment. In this mode, the acquisition software (Analyst TF 1.7, AB Sciex) continuously evaluates the complete scan survey MS data as it collected and triggered the acquisition of MS/MS spectra, depending on preselected criteria. In each cycle, 12 precursor ions whose intensity was greater than 100 were chosen for fragmentation at collision energy (CE) of 30 V (15 MS/MS events with a production accumulation time of 50 msec each). ESI source conditions were set as follows: ion source gas 1, 60 Psi; ion source gas 2, 60 Psi; curtain gas, 35 Psi; source temperature, 650°C; and ion spray voltage floating (ISVF) 5000 V or −4000 V in the positive or negative mode, respectively. Parameters Processing such as retention time alignment, peak discrimination, filtration, alignment matching, and identification were performed by the XCMS package (Scripps Center for Metabolomics and Mass Spectrometry, La Jolla, California).

### GO and KEGG Pathway Analyses of DEGs

The gene expression datasets were filtered from the GEO database, and three gene expression profiles (GSE18606, GSE68004, and GSE73463) were picked among 58 series of KD. All the data were freely available. The differentially expressed genes (DEGs) between KD and normal samples were detected by the GEO2R online analysis tool (https://www.ncbi.nlm.nih.gov/geo/geo2r/), and the P-value and |logFC| were also acquired from the tool. Metascape (https://metascape.org/) was utilized to perform GO annotation and KEGG pathway enrichment analyses of DEGs.

### Reagents

Palmitic acid was purchased from Sigma, BSA (Fatty Acid and IgG-Free, BioPremium), and was provided by Beyotime, and Acetylcysteine (NAC) was bought from MCE. In order to prepare the PA working solution, we first dissolved palmitic acid in DMSO to a storage concentration and then diluted in 1% BSA solution prepared with the corresponding culture medium to achieve a total palmitic acid concentration of 1000 μM. Subsequent different concentrations of palmitic acid were diluted on this sub-basis. This method was established on the Spector’s protocol (Spector, 1986). NAC was dissolved in sterile double distilled water at a concentration of 1 mM after ultrasonic and sterile filtration.

### Cell Culture and Treatment

Human umbilical vein endothelial cells (HUVECs) were bought from Lonza (United States), and Primary Human Aortic Smooth Muscle Cells; Normal (HASMCs) were purchased from ATCC (United States). HUVECs were cultured in an endothelium cell medium (ECM, Sciencell Research Laboratories) with 10% Fetal Bovine Serum (FBS, Gibico) and ECGs, and HASMCs were maintained in Dulbecco’s Modified Eagle Medium (DMEM) with 10% FBS, respectively, at 37°C in a humidified incubator under 5% CO_2_. When performing experiments, cells were seeded in 6-well plates at a density of 3×10^4^ cells/well for HUVECs and 2×10^4^ cells/well for HASMCs. Upon 80% confluence, HUVECs were incubated, after overnight starvation, in corresponding PA or KD serum for 24 h. Meanwhile, HASMCs were treated identically.

### Cell Viability Assays

Cells were resuspended with different concentrations of PA and seeded in 96-well plates at a density of 3 × 103 per well. Cellular cytotoxicity was evaluated using the Cell Counting Kit-8 (CCK-8; Dojindo). After 24 h of treatment, the culture medium was changed to DMEM containing 10 μL per well of Cell Counting Kit-8 (CCK-8, Dojindo) solutions and incubated for a further 2 h at 37°C. The optical density (OD) value was measured at a 490 nm wavelength.

### Western Blot

After treatment, the harvested cells were lysed by RIPA buffer (Byotime) containing a protease and phosphatase inhibitor cocktail (Beyotime). The protein content from each lysate was measured using the BCA Protein Assay Kit (Bio-Rad, Hercules, CA, United States) before loading equal amounts of protein (20 μg/lane) into 12% SDS-PAGE gels. After incubating with 5% non-fat milk for blocking about 2 h, specific antibody for 14h, and secondary antibodies conjugated to horseradish peroxidase for 2 h, the PVDF membrane with ECL developer was performed by the Bio-Rad gel imaging system. The following antibodies were involved in this experiment: p-RB (Abclonal, AP0089), P16(Proteintech, 10883-1-AP), LC3II(CST, 3868), SOD1 (Proteintech, 10269-1-AP), SOD2(Proteintech, 24127-1-AP), and GAPDH (Abways, AB0036).

### SA-β-Gal Staining

The senescent status was demonstrated by the senescence-associated β-galactosidase (SA-β-gal) staining kit (Beyotime). Cells were fixed on the plate and incubated with the mixed staining solutions for 24 h at 37°C.

### Oxidative Stress and ROS Levels

Intracellular ROS levels were evaluated by Dihydroethidium (DHE) fluorescence staining (Beyotime) and Malondialdehyde (MDA) assay kits (TBA method, Nanjing Jianchen Bioengineering Institute). After treatment with either PA or KD serum, cells were incubated with DHE (10 μM) in DMEM at 37°C for 40 min. Fluorescence was detected under excitation at 300 nm and emission at 610 nm. The experiments were performed in the dark conditions. In addition, the supernatant was collected for MDA assays, as per the manufacturer’s instructions. The optical density (OD) value was measured at a 532 nm wavelength.

### Statistical Analysis

The results were expressed as the mean ± SD or median (interquartile range, IQR) for continuous variables according to whether the variables conformed to a normal distribution or as a number (percentage) for categorical variables. The Student’s t-test was used to determine statistical significance for mean ± SD results. In comparison, one-way ANOVA was used for median (IQR) results, and X2 or Fisher’s exact test was used for results expressed as numbers (percentage). Principal component analysis (PCA) and orthogonal projections to latent structures discriminant analysis (OPLS-DA) were applied using SIMCA version 14.0.1 (Umetrics AB, Umea, Sweden). MetaboAnalyst (www.metaboanalyst.ca) was employed for the enrichment pathway analysis. The heatmaps were generated using MeV version 4.6.0. Moreover, the Cytoscape software package version 3.2.0 (National Institute of General Medical Sciences, Bethesda, Maryland) was used to plot the correlation networks. Logistic regression analysis and receiver operating characteristic (ROC) analysis were used to diagnose patients in the CAL and NCAL groups. All statistical analyses were performed using SPSS software version 21.0 (IBM Corp., Armonk, New York). Comparison among experiments groups was analyzed by two-way analysis of variance (ANOVA). A P value of <0.05 or <0.001 was considered statistically significant.

## Results

79 patients were enrolled; none of them had a prior history of KD, and all were clinically diagnosed as typical during the study. There were 40 patients in CAL group and 39 in NCAL group. Based on the AHA KD guidelines (Newburger et al., 2004; Ronai et al., 2016), the average Z score of the CAL group was 4.32 (minimum to 2.5 and maximum to 9.07). Baseline characteristics and drug treatments are presented in Table 1. The high percentage of males enrolled, 72.5 and 71.8%, matched the epidemiology of KD. There were no statistically significant differences between patients in the CAL and NCAL groups except for treatment with persantine. Diverse doses of IVIG based on clinical symptoms were given for 5 days after fever occurred, and as expected, all the patients we chosen responded to IVIG treatment. Fever disappeared 3 days after IVIG treatment in most patients, and fever did not persist in any patient on day 5. The white blood cell (WBC) count and C-reactive protein (CRP) levels and the erythrocyte sedimentation rate (ESR) point towards an inflammatory response. Alanine transaminase (ALT) and aspartate aminotransferase (AST) indicate liver function. Triglycerides (TG), total cholesterol (TC), high density lipoprotein (HDL-C), and low density lipoprotein (LDL-C) indicate distribution.

**TABLE 1:** Baseline characteristics and drug treatment of patients.

	CAL (N = 40)	NCAL (N = 39)	P value
Age, mon	21.7 (8.2–30.9)	23.7 (9.0–40.0)	0.372
Sex, male	29 (72.5%)	28 (71.8%)	0.944
Medication			
IVIG, 2 g/kg*1 d	35 (87.5%)	38 (97.4%)	0.096
IVIG, 1 g/kg*2 days	5 (12.5%)	1 (2.6%)	0.096
Aspirin	38 (95%)	38 (97.4%)	1.000
Persantine	29 (72.5%)	15 (38.5%)	0.002
Complications	25 (62.5%)	25 (64.1%)	0.883
Liver function damage	7 (17.5%)	7 (17.9%)	0.958
Granulocytopenia	8 (20%)	5 (12.8)	0.390
URI	3 (7.5%)	4 (10.3%)	0.972
LRI	8 (20%)	4 (10.3%)	0.228
WBC, 10^9^/L	16.4 ± 6.8	15.8 ± 7.2	0.368
PLT, 10^9^/L	392.7 ± 115.9	374.2 ± 118.1	0.704
CRP, mg/L	75.94 ± 53.6	71.1 ± 51.9	0.395
ESR, mm/h	33.0 ± 12.0	31.8 ± 10.3	0.443
BNP, pg/mL	1505.0 (455.0–3557.5)	833.5 (318.5–2315.0)	0.192
ALT, U/L	50.0 (17.0–115.0)	54.0 (22.0–109.0)	0.734
AST, U/L	35.5 (23.8–51.0)	29.0 (25.0–35.0)	0.294
TG,mmol/L	1.29 ± 0.3	1.3 ± 0.46	0.972
TC,mmol/L	3.56 ± 0.73	3.61 ± 0.63	0.701
HDL-C,mmol/L	1.8 ± 0.75	2.35 ± 0.55	0.043
LDL-C.mmol/L	2 ± 0.84	1.21 ± 0.83	0.000

Data are presented as the mean ± SD, n (%) or medians (interquartile ranges). CAL, coronary artery lesion; NCAL, non-coronary artery lesion; IVIG, intravenous immunoglobulin; URI, upper respiratory infection; LRI, lower respiratory infection; WBC, white blood cell; PLT, platelet; CRP, C-reactive protein; ESR, erythrocyte sedimentation rate; BNP, brain natriuretic peptide; ALT, alanine transaminase; AST, aspartate aminotransferase; TG, triglycerides; TC, total cholesterol; HDL-C, high density lipoprotein; LDL-C, low density lipoprotein.

### Comprehensive Metabolomic Characterization of CALs in KD

**FIGURE 1:**
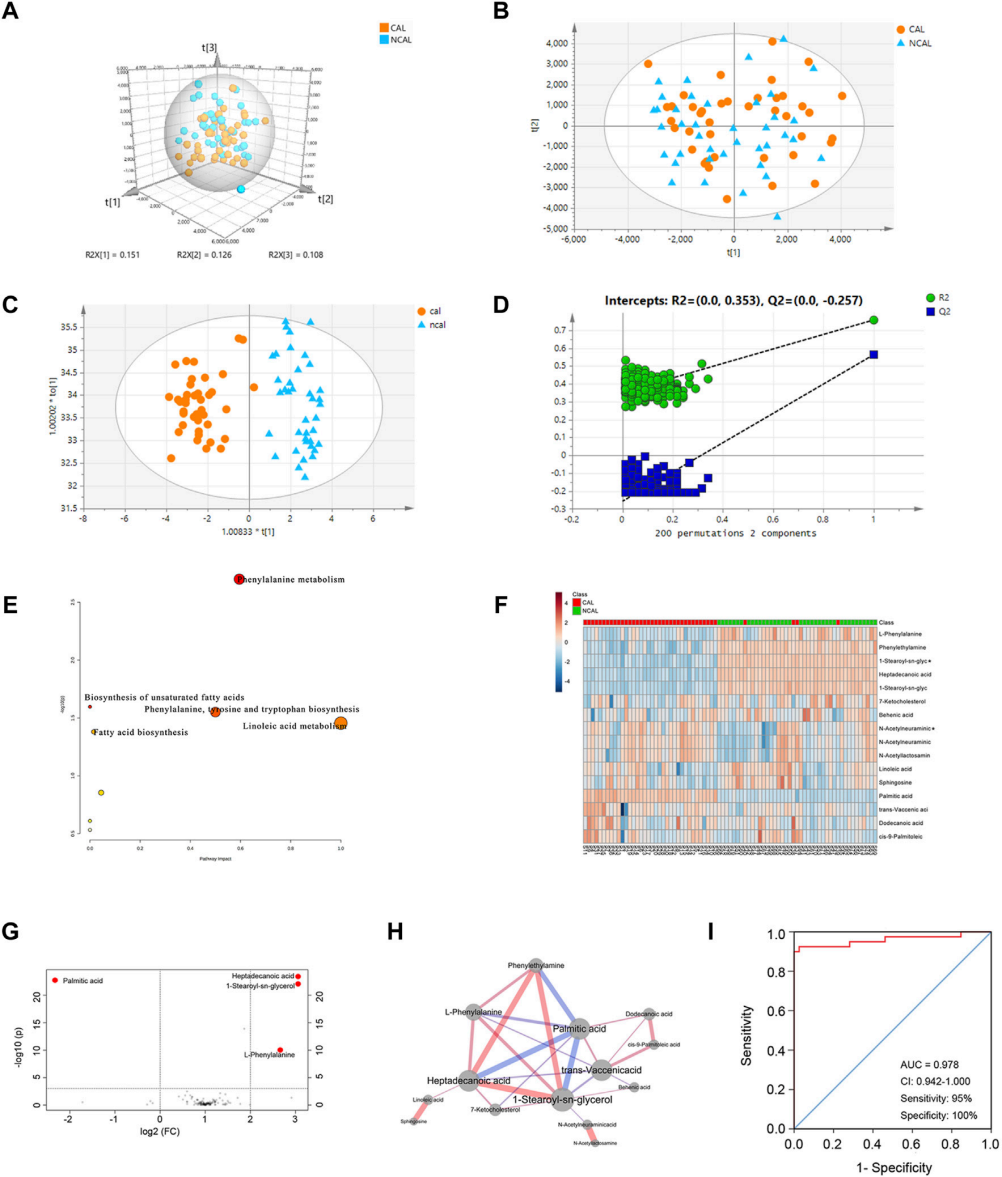
Comprehensive Metabolomic Characterization of CALs in KD. **(A,B)** Principal component analysis (PCA) score plots; **(C)** Orthogonal projections to latent structures discriminant analysis (OPLS-DA) score plots (R^2^Y = 0.867 and Q^2^Y = 0.787); **(D)** Permutation test (*n* = 200) of OPLS-DA; **(E)** Disrupted pathways identified in positive and negative mode; **(F)** Heatmap of the 16 differentially expressed metabolites between the CAL and NCAL groups; red represents a high relative concentration, and blue represents a low relative concentration; **(G)** Volcano plots of different metabolites; **(H)** The network of 14 different metabolites with diverse expressions between the CAL and NCAL groups. The width of each edge represents the correlation coefficient and correlation values; the red edges represent positive correlations, and the blue ones represent negative correlations. Larger nodes represent more connections; **(I)** ROC curve for a panel of biomarkers compared between patients in the CAL and NCAL groups. CAL, coronary artery lesion; NCAL, non-coronary artery lesion; FC, fold change.

The plasma metabolic profiles of patients in the CAL and NCAL groups were measured using UHPLC-QT-MS in both positive and negative modes. After peak alignment and removal of missing values (Supplementary Figure S1), 653 features were detected in positive mode, and 554 features were detected in negative mode. Then we took the features detected in positive and negative mode together for a comprehensive multrivariate statistical analysis. An unsupervised principal component analysis (PCA) model was first used to identify metabolic disturbances (Figure 1A). In the PCA model,the R2 of the three principal components was 0.745, but Q2 was 0.178 lower than 0.4 (Figure 1B), indicating the model’s predicted value is unqualified. Moreover the metabolic profiles between the two groups could not be distinguished (Figure 1B). Therefore, the PCA model failed to identify a difference between the metabolic profiles of patients in the CAL and NCAL groups. To obtain a higher degree of group separation and better understand the variables responsible for classification, supervised OPLS-DA was applied. The metabolic profile was distinguished well by OPLS-DA (Figure 1C), with a cumulative R2Y of 0.867 and Q2Y of 0.787, which were stable and indicated good fitness and prediction. A permutation test (permutation = 200) was applied to validate the statistical significance of the OPLS-DA model. The intercepts were R2 at 0.353 and Q2 at −0.257 (Figure 1D). The OPLS-DA models were considered efficient.

The ions confirmed by MS/MS spectra and mapped in the database with variable importance in the projection (VIP) values >1.0 based on OPLS-DA models were considered to be potential differentially expressed metabolites. Figure 1E describe the pathways affected by the differentially expressed metabolites between patients in the CAL and NCAL groups. The Phenylalanine metabolism, Biosynthesis of unsaturated fatty acids, Phenylalanine, tyrosine and tryptophan biosynthesis, Linoleic acid metabolism, and Fatty acid biosynthesis pathways were altered in the CAL group compared to NCAL one.

To further explore the differentially expressed metabolites, the Student’s t-test was used to identify ion peak intensities with a P-value < 0.05. As a result, 16 metabolites were identified (Table 2). A heatmap of different metabolites is displayed in Figure 1F. Red represents a high relative concentration, whereas blue represents a low relative concentration. The patients could be divided into two groups, with patients with CALs exhibiting a high relative concentration of palmitic acid and a low relative concentration of 1-stearoyl-sn-glycerol, heptadecanoic acid, L-phenylalanine, and phenylethylamine. Due to the complex relationships among metabolites in the human body, a correlation analysis could probably reveal the critical nodes in the network. Therefore, a metabolite correlation matrix using the 16 metabolites was generated based on Pearson’s correlation coefficients. A P value <0.05 was used to construct the network (Figure 1H). The red edges represent positive correlations. Conversely, the blue ones represent negative correlations. The width of each edge represents the correlative coefficient value, with larger nodes representing more connections. The network exhibited that palmitic acid, 1-stearoyl-sn-glycerol, heptadecanoic acid, and phenylethylamine were the crucial metabolites in KD patients with CALs.

**TABLE 2:** Differentially expressed metabolites.

Differential metabolite	Mass-to-charge ratio	Retention time (sec)	Fold change	P value
Higher concentration in CAL				
Palmitic acid	274.273	52.950	9.879	<0.001
N-Acetylneuraminic acid^a^	290.086	514.804	1.554	0.003
Dodecanoic acid	218.210	92.196	1.499	0.009
cis-9-Palmitoleic acid	296.258	36.189	1.361	0.036
N-Acetylneuraminic acid	292.102	515.098	1.322	0.019
N-Acetyllactosamine	366.139	515.061	1.259	0.030
trans-Vaccenic acid	265.251	35.104	1.223	0.002
Lower concentration in CAL				
1-Stearoyl-sn-glycerol^a^	417.319	35.321	0.162	<0.001
1-Stearoyl-sn-glycerol	341.305	35.328	0.239	<0.001
Heptadecanoic acid	288.289	73.946	0.244	<0.001
L-Phenylalanine	331.165	254.527	0.316	<0.001
Phenylethylamine	281.137	36.312	0.549	<0.001
Behenic acid	358.366	139.662	0.608	0.046
Linoleic acid	298.273	111.232	0.734	0.010
7-Ketocholesterol	461.362	34.234	0.795	0.011
Sphingosine	300.289	109.074	0.800	0.040

a Metabolites were confirmed by negative-mode analysis.

To identify potential biomarkers for CALs in patients with KD, a cut-off P-value < 0.001 and FC (fold change) > 2 were used in both positive and negative mode. The volcano plots of these metabolites are shown in Figure 1G. Four metabolites, 1-stearoyl-sn-glycerol, palmitic acid, heptadecanoic acid, and phenylethylamine, were considered as potential biomarkers. Furthermore, logistic regression analysis of each biomarker was used to draw the ROC curve. For the ROC curve shown in Figure 1I, the area under the curve (AUC), sensitivity, and specificity was 0.978, 95%, and 100%, respectively. This panel of biomarkers was robust enough to distinguish between KD patients in the CAL and NCAL groups.

### Functional Enrichment Analyses of DEGs Between the KD and Normal Groups

**FIGURE 2:**
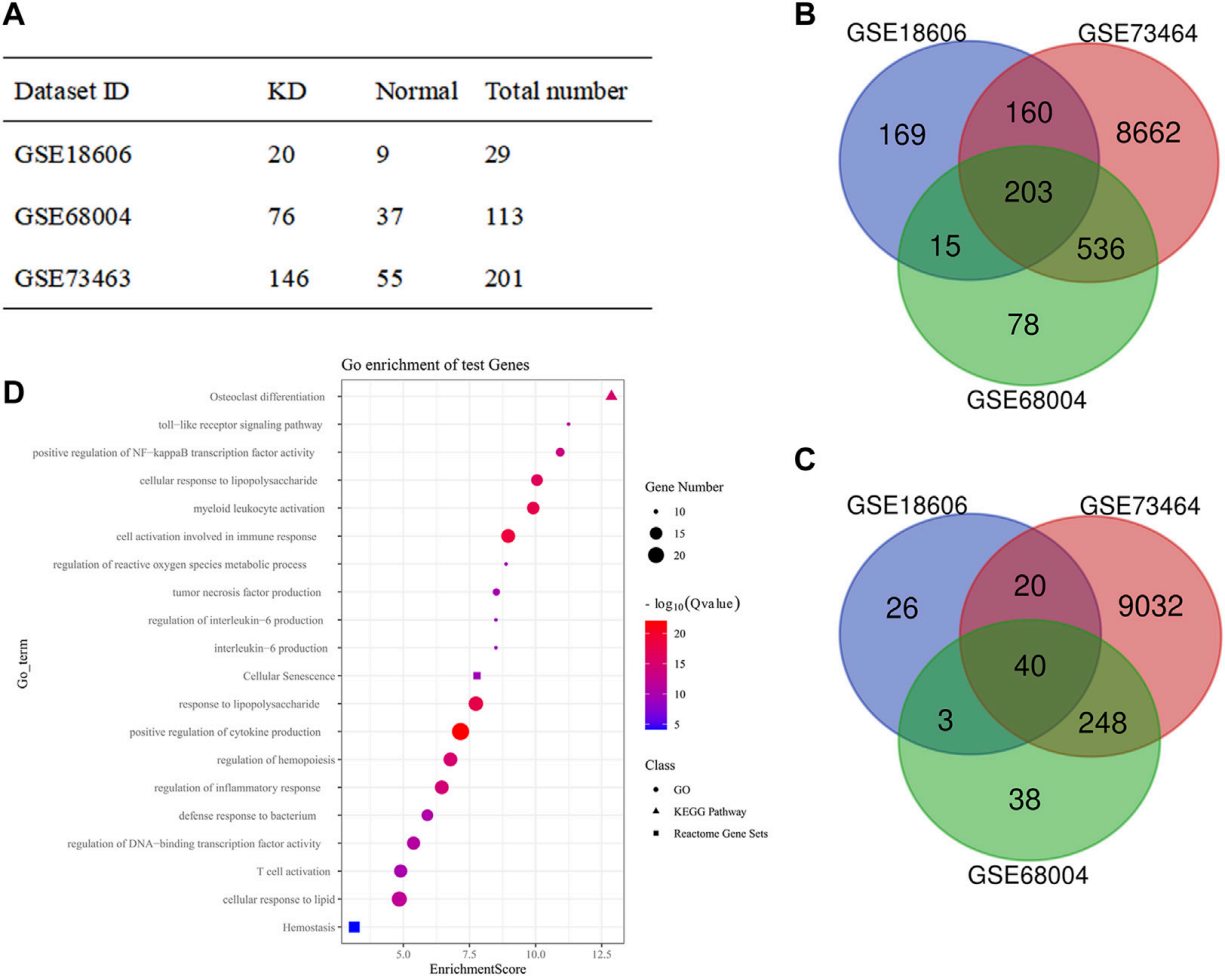
Functional enrichment analyses of DEGs between KD and Normal group. **(A)** Statistical table from the three microarray databases searched in the GEO database; **(B,C)** The Venn diagram of the intersection among DEGs from all three GEO data sets: **(B)** Upregulated genes; **(C)** Downregulated genes; **(D)** Bubble diagram from the GO terms, KEGG pathway, and Reactome pathway of significantly enriched DEGs. DEG, differentially expressed gene; GEO, gene expression omnibus.

Due to a lack of datasets concerning the gene expressions between CALs and NCALs, we chose three gene expression series (GSE18606, GSE68004, and GSE73463) to analyze the functional enrichment of DEGs between the KD and normal groups in this study. Among these three groups, there were a total of 242 KD samples and 101 normal ones (Figure 2A). Based on the standard of *p* < 0.05 and |logFC| ≥ 1.0, the upregulated genes and downregulated genes were identified by comparing KD specimens with normal specimens among these three groups. After that, the intersections among these three DEG profiles were carried out by Venn analysis (Figures 2B,C). Overall, 23 upregulated genes and 149 downregulated genes were significantly differentially expressed.

The functional enrichment analyses, including GO function, KEGG, and Reactome pathway enrichment analyses of DEGs, were performed *via* Metascape. The top 20 GO terms were ranked by enrichment scores, demonstrating that DEGs were mainly enriched in immune response, inflammatory response, reactive oxygen species metabolic process, and so forth (Figure 2D). Notably, cellular senescence and response to lipid were also enriched, which are seldomly discussed in KD and the core roles of our subsequent studies.

### Palmitic Acid Affected Cellular Activities by Inducing Autophagy and Cellular Senescence

**FIGURE 3:**
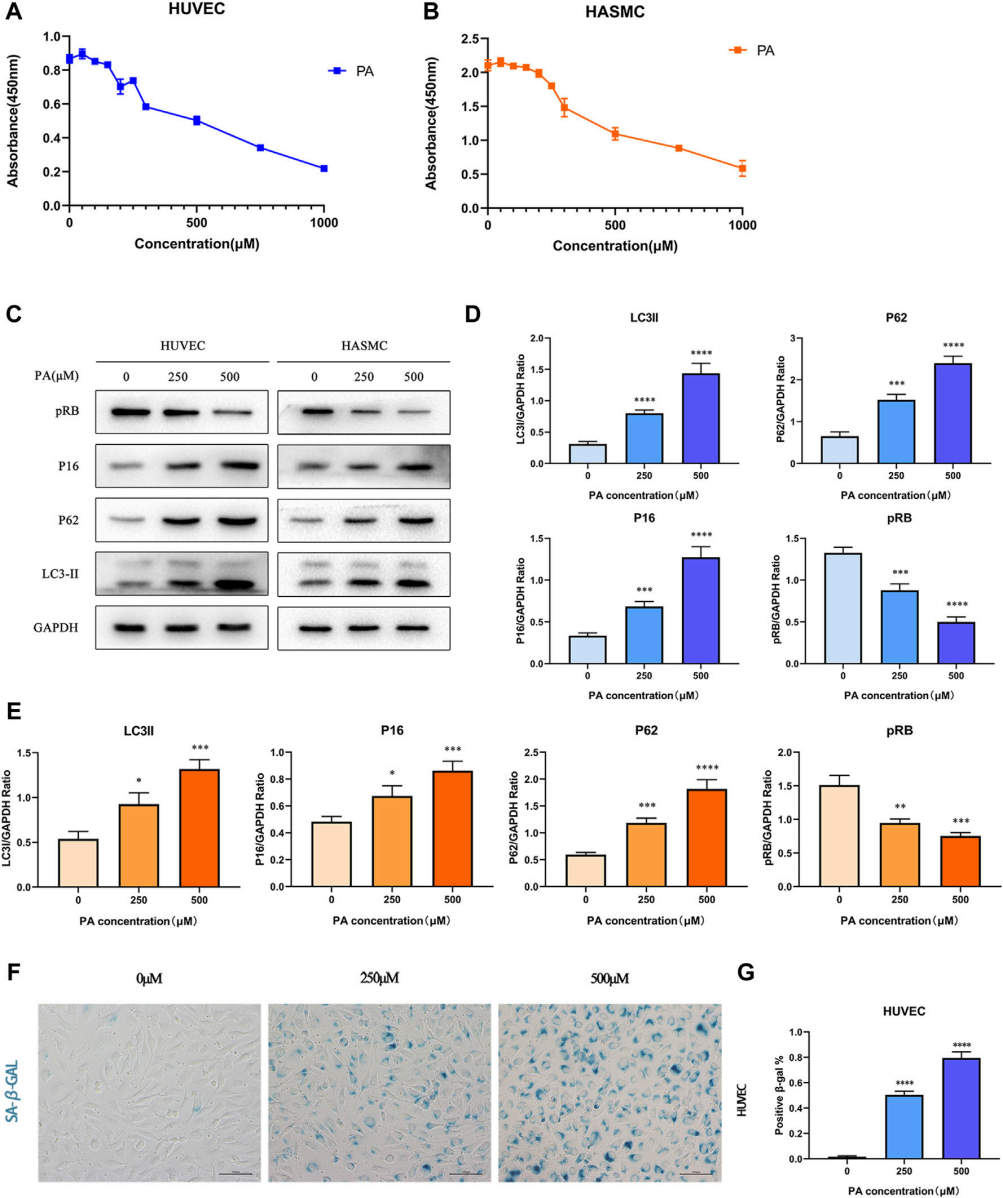
PA affected cell activity by inducing autophagy and cell senescence **(A,B)** HUVECs and HASMCs were treated with diffferent concentrations of PA. CCK8 assay established that cellular activities was inhibited; **(C–E)** HUVECs and HASMCs were treated with PA at the concentrations of 250 and 500 μM. Control groups were treated with 1%BSA. Incubated for 24 h, total proteins were probed for P16, pRB, P62 and LC3II proteins. GAPDH was used as the loading control **(C)**; Relative protein expression levels of P16, pRB, P62, and LC3II compared with GAPDH in HUVECs **(D)**; Relative protein expression levels of P16, pRB, P62, and LC3II compared with GAPDH in HASMCs **(E)**; **(F,G)** HUVECs were treated with PA under the concentrations of 250 and 500 μM. The presence of SA-β-gal activity in HUVECs indicated cellular senescence was detected **(F)**; Quantification of SA-β-gal staining. The number of SA-β-gal positive cells under 250 and 500 μM PA concentrations were significantly higher than in the control group **(G)**. PA, Palmitic acid. (*n* = 6; Data shown as Mean ± SEM; *p < 0.05, **p < 0.01, ***p < 0.001, ****p < 0.0001 compared to control groups).

Based on previous studies, PA can induce oxidative stress and apoptosis as a saturated fatty acid (Yu et al., 2020; Lee et al., 2021). In order to explore the role of PA in coronary artery diseases of KD, we selected 2 cell lines commonly used in KD research: HUVECs and HASMCs, and performed the CCK-8 assay to detect the cytotoxicity of PA in both cell lines. As shown in Figures 3A,B, PA inhibited the activity of HUVEC cells in a palmitic concentration-dependent manner. Interestingly, cellular activities were significantly reduced at a concentration of about 250 μM. Likewise, cellular activities were reduced to 50% of the control group at a concentration of 500 μM. This phenomenon was observed in HASMC cells as well. Then, we chose two concentration gradients of 250 and 500 μM to study further the effects of PA on the 2 cell lines. By identifying multiple indicators in both cell lines (Figures 3C–E), it was found that with an increase of PA concentration, the expression levels of autophagy representative protein P62 and LC3II similarly increased, while the expression of cell senescence symbols p16 increased and pRB decreased. This result suggests that PA may cause cellular injury by inducing autophagy and cellular senescence. According to the GEO analysis, cellular senescence was enriched in the KD group. Thus, we further performed SA-β-GAL staining under the concentrations of 250 and 500 μM within the 2 cell lines. The presence of SA-β-GAL only demonstrated aggravated cellular senescence in HUVECs (Figures 3F,G), which could barely be detected in HASMCs (Supplementary Figure S2).

### Palmitic Acid Aggravated Cellular Senescence of Endothelial Cells in Kawasaki Disease

**FIGURE 4:**
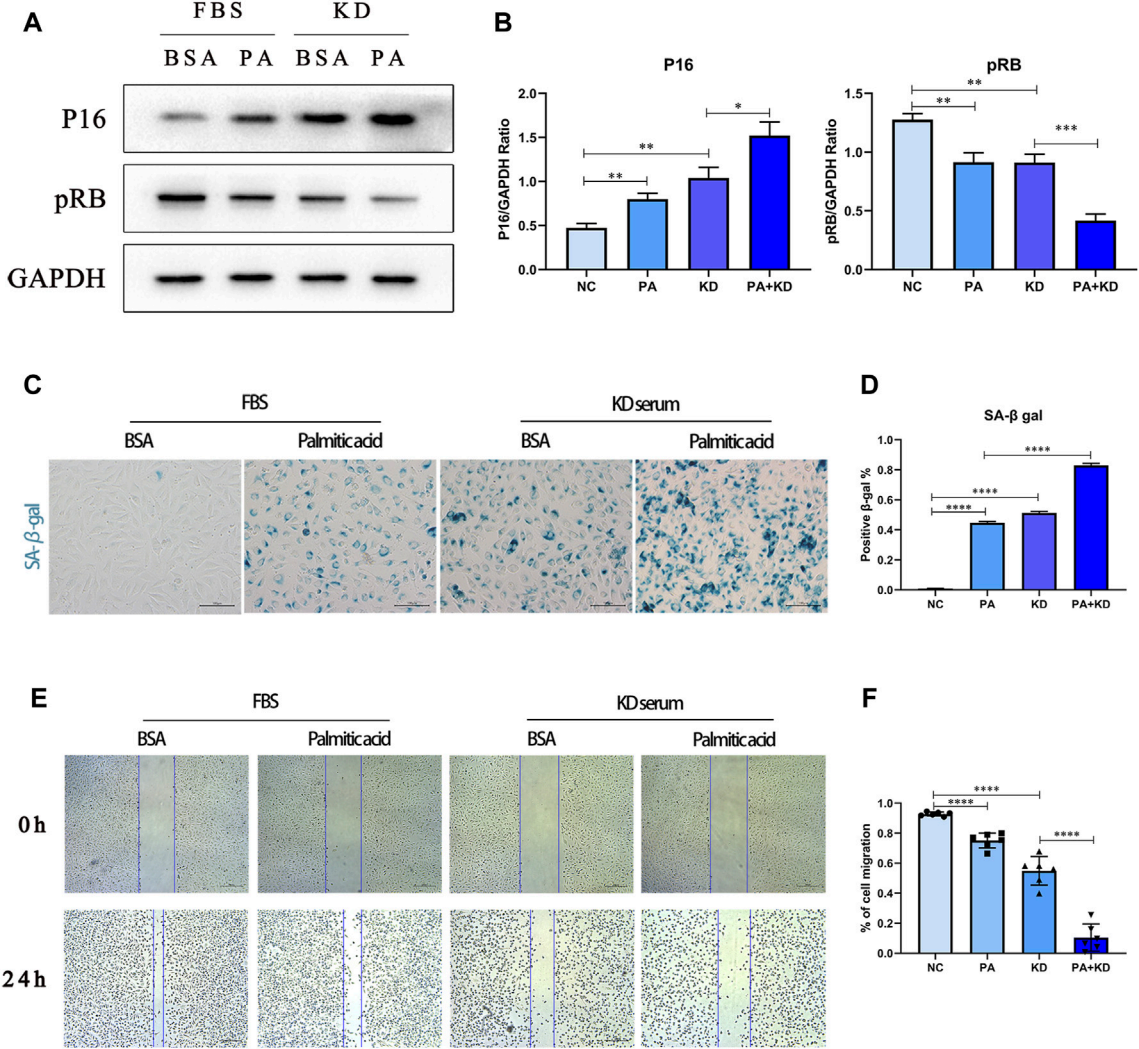
PA aggravated cellular senescence of endothelial cells in Kawasaki disease. **(A,B)** HUVECs were co-treated with 500 μM PA and 10% KD serum (line 4), or only PA (line 2), or only KD (line 3). Control groups were co-treated with 1%BSA and 10%FBS (line 1). Incubated for 24 h, total proteins were probed for P16 and pRB proteins; Relative protein expression levels of P16 and pRB compared with GAPDH; **(C,D)** HUVECs were treated with 500 μM PA or 10%KD serum or both compared with control group. The staining of SA-β-gal activity indicated PA aggravated cell senescence in HUVECs **(C)**; Quantification of SA-β-gal staining **(D)**; **(E,F)** HUVECs were treated with 500 μM PA or 10%KD serum or both, and compared with the control group. Endothelial cell function was detected by cell migration **(E)**; the bar graph represented the quantification of the scratch assay **(F)**. (*n* = 6; Data shown as Mean ± SEM; *p < 0.05, **p < 0.01, ***p < 0.001, ****p < 0.0001).

We further tested whether palmitic acid worsened endothelial cell senescence in KD. First, we tested the expression of cellular senescence-associated proteins cyclin-dependent kinase inhibitor 2A (P16) and Phospho-RB-S811 (pRB), as well as SA-β-gal with specific staining for cellular senescence. Our results showed that compared with the BSA control group, the expression level of P16 increased after stimulation by KD serum. In contrast, the expression level of pRB decreased, implying that KD serum also induced endothelial cell senescence. Based on that, the addition of PA further exacerbated cellular senescence. (Figures 4A,B). At the same time, SA-β-gal staining also verified this point (Figures 4C,D). Compared with the BSA control group, the KD group had more stained cells, and the positive spots increased significantly after the addition of PA treatment.

At the same time, when endothelial cells become senescent, some studies have shown that endothelial cell function is also hindered, and proliferative and migrative abilities are weakened. Therefore, we used the endothelial cell scratch test to analyze the changes in migration ability (Figures 4E,F). Compared with the BSA control group, a single treatment of PA or KD serum stimulation reduced the migration ability of endothelial cells. Furthermore, co-treatment with PA and KD serum further weakened the migration ability compared with the KD serum group. In summary, KD serum can induce endothelial cells transferred to a status of cell senescence. Moreover, PA aggravated this transforming process and was accompanied by a further aggravation of endothelial cell dysfunction.

### Palmitic Acid Also Increases Intracellular ROS Accumulation of Endothelial Cells in KD

**FIGURE 5:**
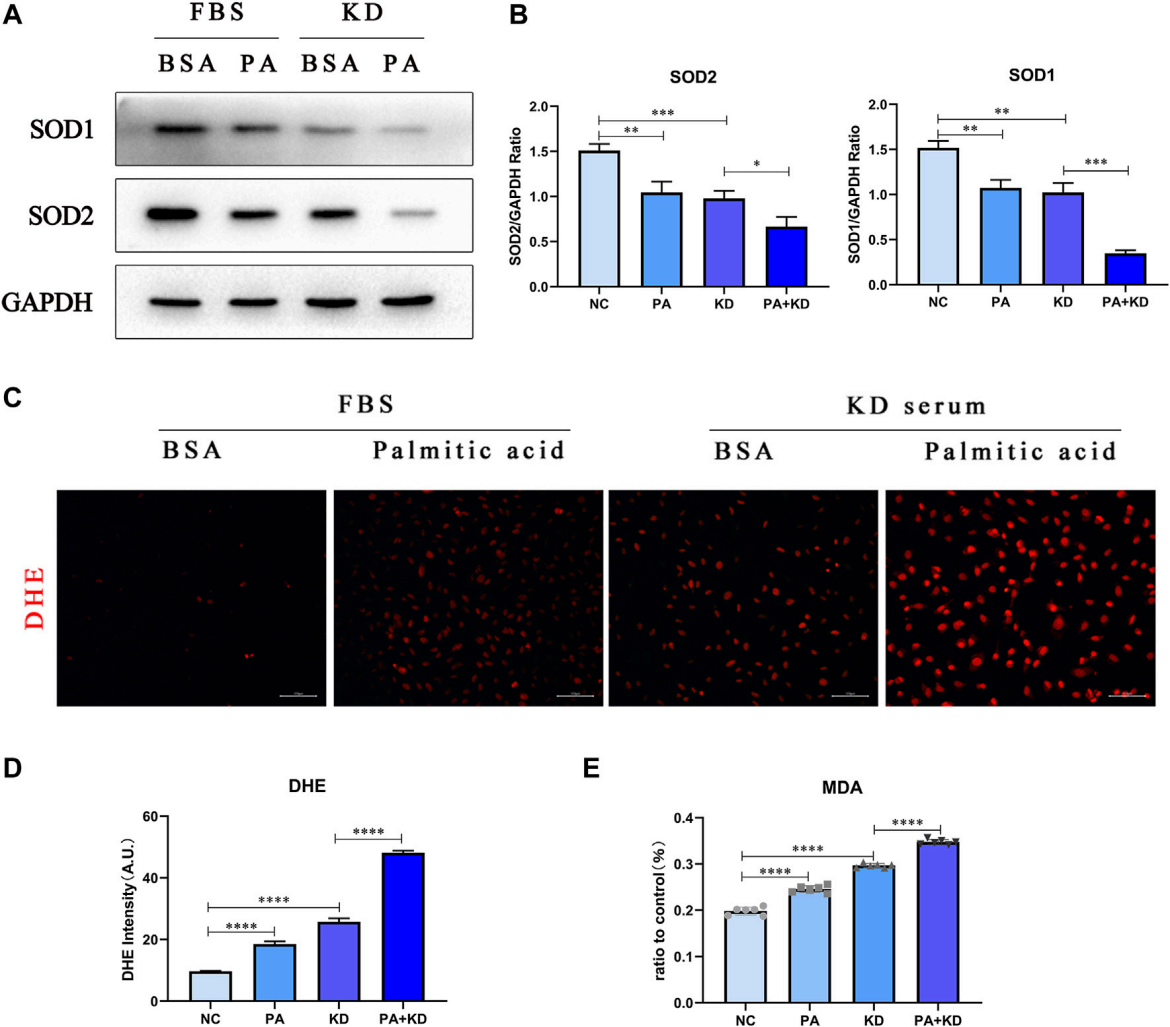
PA also increased intracellular ROS accumulation in endothelial cells in KD. **(A,B)** HUVECs were co-treated with 500 μM PA and 10% KD serum (line 4), PA only (line 2), or KD only (line 3). Control groups were co-treated with 1% BSA and 10% FBS (line 1). Incubated for 24 h, total proteins were probed for SOD1 and SOD2 proteins; Relative protein expression levels of SOD1 and SOD2 compared with GAPDH; **(C,D)** Immunofluorescent images of DHE-stained HUVECs after treatment for 24 h **(C)**. The quantitative data from Immunofluorescent images **(D)**; **(E)** MDA tests from the supernatant of HUVECs after treatment for 24 h (*n* = 6; Data shown as Mean ± SEM; *p < 0.05, **p < 0.01, ***p < 0.001, ****p < 0.0001).

Based on the GEO analysis, the reactive oxygen species metabolic process was also enriched in the KD group. Therefore, we further explored whether PA, a metabolite enriched in KD metabolites, activated the production of reactive oxygen species in endothelial cells. We used immunofluorescence staining of superoxide anion probe DHE to detect the endothelial cells pre-stained with DHE and observed that PA or KD serum treated alone increased ROS generation. At the same time, the co-treatment of PA and KD serum further increased the production of ROS compared with the group treated with KD serum alone (Figures 5C,D). In addition to the formation of excessive ROS, the imbalance of oxidative stress in the body may also be caused by an impaired active oxygen scavenging mechanism. As a member of the active oxygen scavenger family, superoxide dismutase (sod) is the first line of defense against ROS production. Sods in humans mainly include SOD1/2/3, and SOD1 and SOD2 are located intracellularly. Therefore, we primarily detected SOD1 and SOD2 proteins and found that after being treated with PA or KD serum alone, the production of SOD1/2 decreased. Meanwhile, compared with the KD group, co-treatment of PA and KD serum further impeded the production of sod1/2, which proved that PA attenuated the production of superoxide dismutase in endothelial cells after KD serum treatment (Figures 5A,B).

Excessive production of oxygen free radicals will further attack the polyunsaturated fatty acids in the cell membrane, trigger lipid peroxidation, and form lipid peroxides, which will trigger the cross-linking and polymerization of macromolecular nucleic acids and proteins, resulting in cytotoxicity. Therefore, we chose one of the lipid peroxides, MDA, to detect the cytotoxicity caused by reactive oxygen species, proving that PA significantly aggravated the cytotoxicity caused by reactive oxygen species in endothelial cells after KD serum treatment (Figure 5E).

### Palmitic Acid Aggravates Cellular Senescence by Mediating ROS Production

**FIGURE 6:**
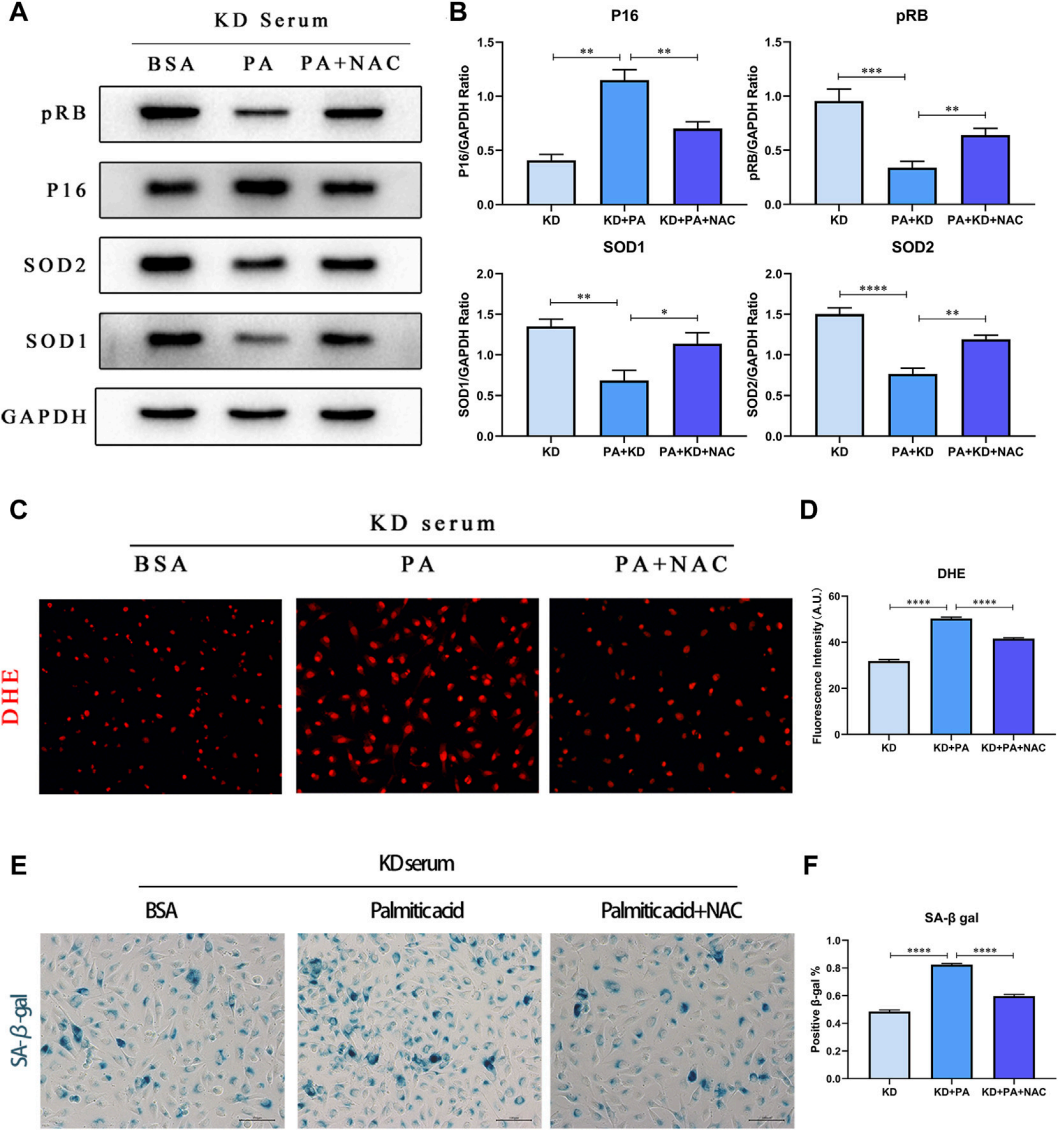
PA aggravates cell senescence by mediating ROS production. **(A,B)** HUVECs were co-treated under 10% KD serum with 500 μM PA (line 2) or 500 μM PA and NAC (line 3), compared with control groups co-treated with 1% BSA and 10% KD serum (line 1). Incubated for 24 h, total proteins were probed for P16, pRB, SOD1, and SOD2 proteins **(A)**; Relative protein expression levels of P16, pRB, SOD1, and SOD2, compared with GAPDH **(B)**; **(C,D)** Immunofluorescent images of DHE-stained HUVECs after treatment for 24 h **(C)**. The quantitative data from immunofluorescent images **(D)**; **(E,F)** The staining of SA-β-gal activity indicated that decreased ROS production by NAC lessened cellular senescence induced by PA in HUVECs **(C)**; Quantification of SA-β-gal staining. (*n* = 6; Data shown as Mean ± SEM; *p < 0.05, **p < 0.01, ***p < 0.001, ****p < 0.0001; scale bar = 100 μm).

As the research on the mechanism of aging in KD is unclear, currently recognized cellular senescence triggers include DNA damage, shortening and damage of telomerase, and activation of oncogenes. Reports have outlined that reactive oxygen species not only attack lipids but also damage mitochondria and nuclear DNA. Therefore, we chose acetylcysteine (NAC), an inhibitor of reactive oxygen species, to further verify whether PA mediates the increase in ROS production and leads to cellular senescence in the KD model. As displayed in Figures 6A–D, the detection of proteins SOD1/2 and DHE probe staining indicated that the addition of NAC decreased ROS production and increased the expression level of superoxide dismutase. On this basis, we detected pRB and P16 proteins, while senescence-specific SA-β-gal staining showed that under the premise of inhibiting the production of reactive oxygen species, the performance of senescence was also reversed (Figures 6E,F).

**FIGURE 7:**
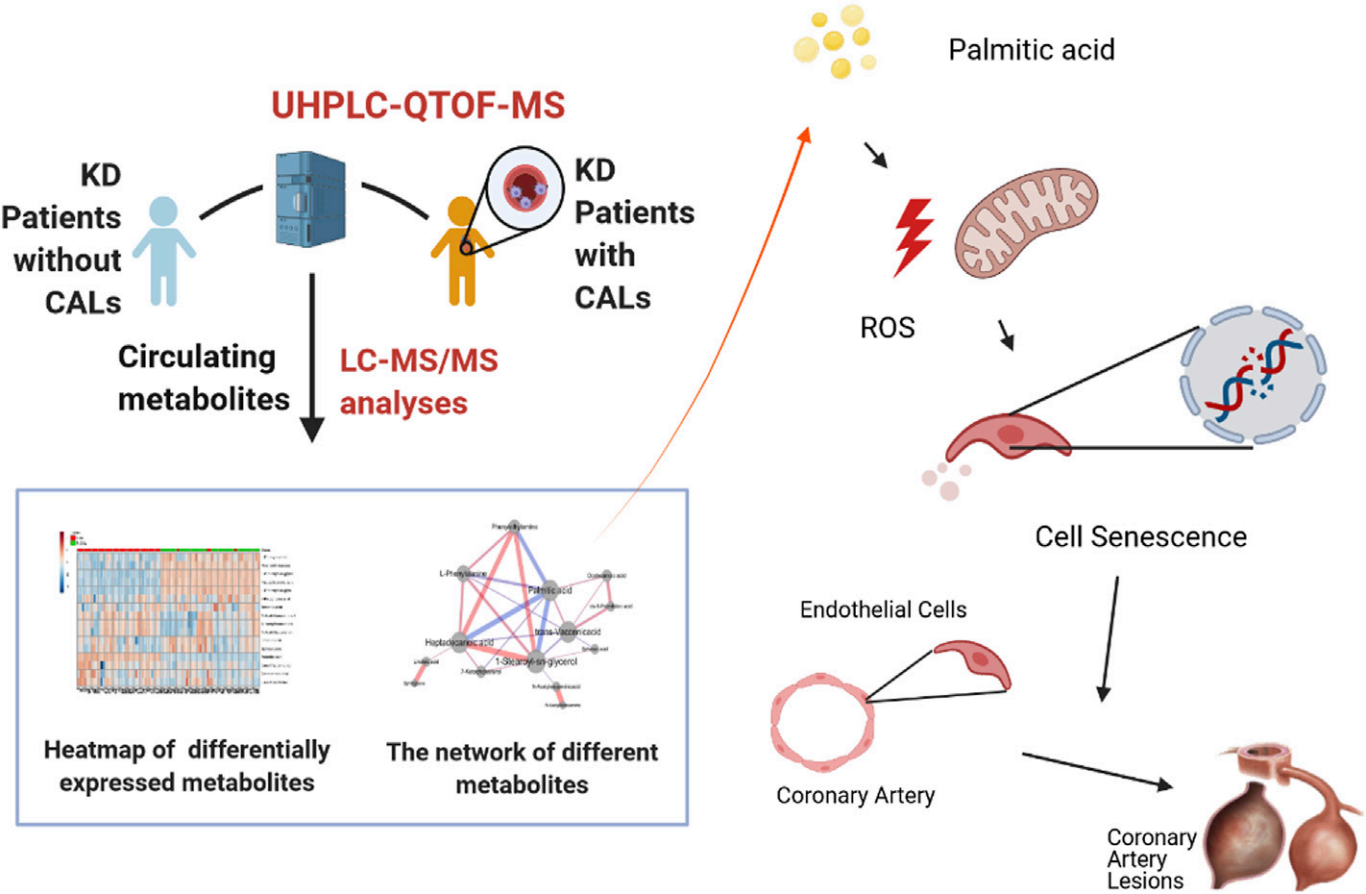
Palmitic acid, a critical metabolite, aggravates cell senescence through reactive oxygen species generation in Kawasaki disease.

In summary, we analyzed the differences in plasma metabolites between the CAL and NCAL groups *via* metabolomics methods. We identified four primary bile acid biosynthetic metabolisms in the coronary artery injury group, namely phenylalanine metabolism and fatty acid biosynthesis. The destruction of the pathway and the differential expression of metabolites in 14, PA showed the most considerable fold change. In subsequent experiments, we further proved that in the cell model of KD, PA further aggravated endothelial cell senescence by increasing the production of reactive oxygen species in endothelial cells, thereby worsening cellular damage (Figure 7). Therefore, cellular senescence may be one of the mechanisms of vascular endothelial injury in KD. PA may be a promising biomarker and potential therapeutic target for predicting the occurrence of CAL in KD patients.

## Discussion

As the first study to identify the differences in circulating metabolites between the CAL and NCAL groups with the application of UHPLC-QTOFMS, a key result of metabolomics was PA showing the most massive fold change among all the significantly differently expressed metabolites. Alongside the metabonomics findings, we further demonstrated the potential role played by PA in the pathogenesis of KD patients with CALs. Our results signaled that PA exacerbates endothelial cell senescence by increasing the production of reactive oxygen species in endothelial cells, and cellular senescence may be one of the mechanisms of coronary artery lesions in KD.

The metabolic profiles demonstrated significant pattern differences between patients in the CAL and NCAL groups in the acute phase. Moreover, the alterations in metabolites suggested that several metabolic pathways were disrupted in KD patients with CALs, including the Phenylalanine metabolism, Linoleic acid metabolism, and Fatty acid biosynthesis pathways. A panel of biomarkers showed excellent predictive values in distinguishing between KD patients with CALs and those without CALs. PA, heptadecanoic acid and 1-stearoyl-sn-glycerol are fatty acids. Among these three fatty acids, PA had the highest fold change (FC = 9.88) and high relative connections in patients with CALs compared with patients in the NCAL group. At the same time, the other two showed moderate fold changes (FC = 4.09 for heptadecanoic acid, and FC = 4.17 for 1-stearoyl-sn-glycerol) and low relative connections in patients with CALs. Therefore, we speculate that PA is involved in the development of CALs in patients with KD.

Numerous studies have affirmed that PA can accelerate artery damage. Cheng et al. (2017) found that palmitic acid could inhibit aortic vascular smooth muscle cell proliferation, enhance autophagy flux and promote vascular remodeling by elevating MMP-2 and MMP-9. In low-density lipoprotein receptor-deficient mice, palmitic acid could increase thoracic aorta atherosclerosis and inflammatory factors such as IL-6 and IL-1 beta (Lu et al., 2017). Furthermore, Hendgen-Cotta et al. (2017) demonstrated that palmitic acid was preferentially incorporated in triglycerides in mice lacking myoglobin and that myoglobin protected against cardiac lipotoxicity by suppressing this incorporation. As described above, most of these studies were focused on atherosclerosis, which is associated with disrupted fatty acid metabolism (Cheung et al., 2004; Mitani et al., 2005; Cheung et al., 2007; McCrindle et al., 2007; Niboshi et al., 2008). Reviewing the latest data, although there is no conclusive consensus about whether a history of KD will provoke premature atherosclerosis in patients, the risk factors for atherosclerosis have already been proved to exist in patients with a history of KD history, and there should be pathophysiological and genetic similarities between those two.

Initial studies have imposed that as one of the characteristic histological features, endothelial cell senescence is closely related to atherosclerosis. Honda et al. (2021) identified a positive correlation of EC senescence with the development of atherosclerosis by utilizing unique EC-specific progeroid mice. Correspondingly, Fukazawa et al. (Fukazawa et al., 2007) detected increased senescence-associated β-gal activity in the luminal surface of the endothelium, which is consistent with our results. Furthermore, GO enrichment analysis found that the upregulated genes were enriched in the cellular senescence and regulation of reactive oxygen species metabolic process. We confirmed that the accelerating ROS in endothelial cells co-treated with KD serum and PA could exacerbate the cellular senescence, which may be a part of the mechanisms of cell senescence in endothelium cells.

Considering the severe effects of CALs in patients with KD, we advocate a new method to predict the risk of CALs in the early stage of the disease, which contributes to these patients’ treatment. 1-Stearoyl-sn-glycerol, palmitic acid, heptadecanoic acid, and phenylethylamine had sufficient power to distinguish between patients in the CAL and NCAL groups individually (the AUC values ranged from 0.913 to 0.956). Our findings may also provide a novel vision for elucidating the pathogenesis of KD patients with CALs. In summary, we will further explore whether the other three biomarkers, excluding PA can exacerbate CALs in KD patients and verify our findings in CAWS models in the next stage of our study. In addition, the triggering factors of cellular senescence in endothelial cells will be further analyzed.

## Data Availability

The original contributions presented in the study are included in the article/Supplementary Material, further inquiries can be directed to the corresponding authors.

## Supplementary Material

Supplementary Figure S1 The typical peak intensity chromatograms of quality control samples under negative and positive modes. **(A)** negative mode; **(B)** positive mode. These two figures illustrated the stability and repeatability of detection systems.

Supplementary Figure S2 The presence of SA-β-gal under PA treated in HASMCs. HASMCs were treated with PA under the concentrations of 250 and 500 μM. The staining of SA-β-gal activity in HASMCs seldom indicated that cellular senescence was detected.
