# Supplementary figures and images for: Palmitic Acid, A Critical Metabolite, Aggravates Cellular Senescence Through Reactive Oxygen Species Generation in Kawasaki Disease
Source: Front Pharmacol. 2022 Mar 23;13:809157. doi: 10.3389/fphar.2022.809157 (PMC8983937; doi:10.3389/fphar.2022.809157)

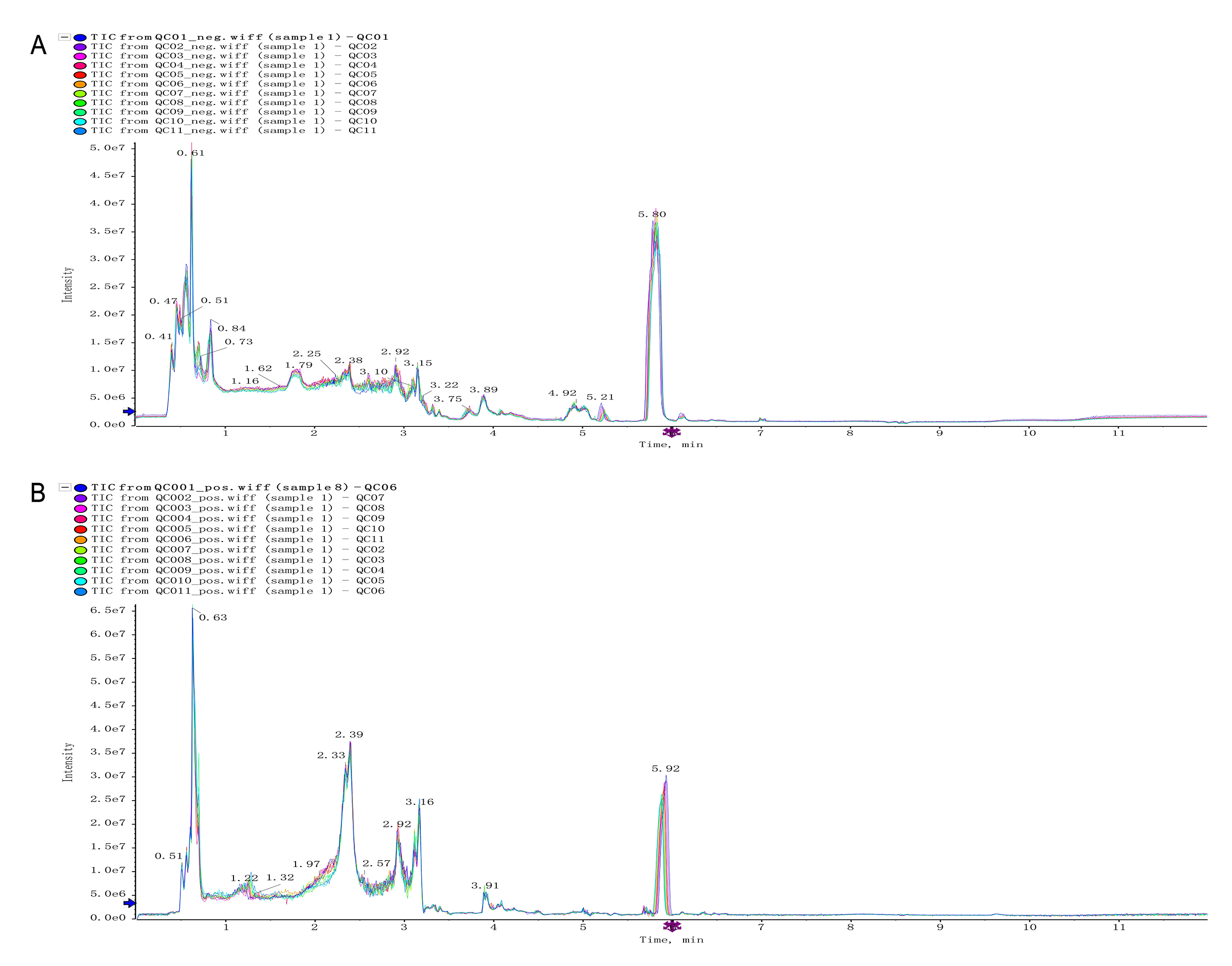

Supplement: Supplementary file 4 [file Image2.TIF]

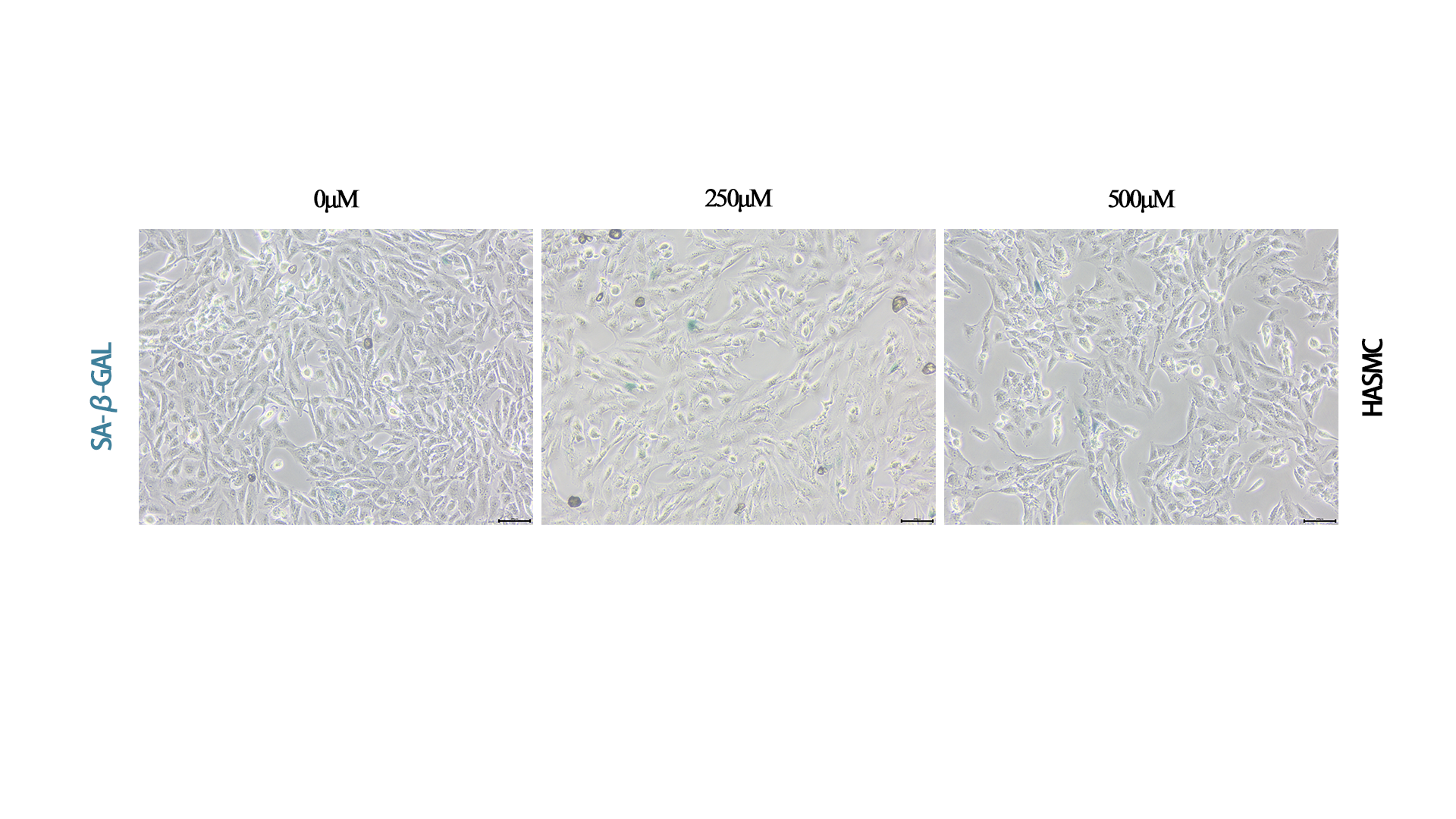

Supplement: Supplementary file 5 [file Image1.TIF]
